# Molecular signatures (unique proteins and conserved indels) that are specific for the epsilon proteobacteria (*Campylobacterales*)

**DOI:** 10.1186/1471-2164-7-167

**Published:** 2006-07-04

**Authors:** Radhey S Gupta

**Affiliations:** 1 Department of Biochemistry and Biomedical Sciences, McMaster University, Hamilton, L8N 3Z5, Canada

## Abstract

**Background:**

The epsilon proteobacteria, which include many important human pathogens, are presently recognized solely on the basis of their branching in rRNA trees. No unique molecular or biochemical characteristics specific for this group are known.

**Results:**

Comparative analyses of proteins in the genomes of *Wolinella succinogenes *DSM 1740 and *Campylobacter jejuni *RM1221 against all available sequences have identified a large number of proteins that are unique to various epsilon proteobacteria (*Campylobacterales*), but whose homologs are not detected in other organisms. Of these proteins, 49 are uniquely found in nearly all sequenced epsilon-proteobacteria (viz. *Helicobacter pylori *(26695 and J99), *H. hepaticus*, *C. jejuni *(NCTC 11168, RM1221, HB93-13, 84-25, CF93-6, 260.94, 11168 and 81-176), *C. lari, C. coli, C. upsaliensis, C. fetus*, *W. succinogenes *DSM 1740 and *Thiomicrospira denitrificans *ATCC 33889), 11 are unique for the *Wolinella *and *Helicobacter *species (i.e. *Helicobacteraceae *family) and many others are specific for either some or all of the species within the *Campylobacter *genus. The primary sequences of many of these proteins are highly conserved and provide novel resources for diagnostics and therapeutics. We also report four conserved indels (i.e. inserts or deletions) in widely distributed proteins (viz. B subunit of exinuclease ABC, phenylalanyl-tRNA synthetase, RNA polymerase β '-subunit and FtsH protein) that are specific for either all epsilon proteobacteria or different subgroups. In addition, a rare genetic event that caused fusion of the genes for the largest subunits of RNA polymerase (*rpoB *and *rpoC*) in *Wolinella *and *Helicobacter *is also described. The inter-relationships amongst *Campylobacterales *as deduced from these molecular signatures are in accordance with the phylogenetic trees based on the 16S rRNA and concatenated sequences for nine conserved proteins.

**Conclusion:**

These molecular signatures provide novel tools for identifying and circumscribing species from the *Campylobacterales *order and its subgroups in molecular terms. Although sequence information for these signatures is presently limited to *Campylobacterales *species, it is likely that many of them will also be found in other epsilon proteobacteria. Functional studies on these proteins and conserved indels should reveal novel biochemical or physiological characteristics that are unique to these groups of epsilon proteobacteria.

## Background

The epsilon (ε-) proteobacteria comprise one of the five Classes within the phylum Proteobacteria [1-4]. These bacteria inhabit a wide variety of ecological niches ranging from gastrointestinal tracts of animals to water reservoirs, sewage, oil-field community and deep-sea hydrothermal vents [2,5-10]. Recent studies show that ε-proteobacteria comprise a significant proportion of the microbial population in deep-sea hydrothermal vents where, because of their ability to carry out different types of metabolism using a variety of alternate electron donors (e.g. H_2_, formate, elemental sulfur, sulfide, thiosulfate) and acceptors (e.g. sulfite, elemental sulfur, nitrate), they play important role in carbon, nitrogen and sulfur cycles [7,9-13]. A great deal of interest in these bacteria stems from the fact that many of these species are host-associated (*Helicobacter, Campylobacter, Wolinella*) and comprise important human and animal pathogens [14-16]. Of these bacteria, *Helicobacter pylori *is the causative agent for gastric and peptic ulcers [17,18] and infections with this and the related species *H. hepaticus *are important predisposing factors in gastric cancers in humans and liver cancers in rodents [16,19,20]. *Campylobacter jejuni *and *C. coli *are the most common causes of food-born illnesses such as diarrhea worldwide [15,21]. *C. jejuni *infection can also lead to the neuromuscular disease Guillain-Barre syndrome [15,21,22], which causes weakness and paralysis of muscles. In contrast to the pathogenic nature of *Helicobacter *and *Campylobacter*, *Wolinella succinogenes *is a commensal in the gastrointestinal tract of cattle and it is not known to cause any illness in either animals or humans [2,5,14,23]. In addition to the host-associated species, many free-living members which include chemolithotrophic and autotrophic bacteria (e.g., *Thiomicrospira denitrificans*, *Arcobacter*, *Caminibacter, Nautilia, Thiovulum*) also form part of the ε-proteobacterial group [4,6,8,10,12,24].

The ε-proteobacteria are presently distinguished from other bacteria based their branching in the 16S rRNA trees [2,4-6]. Although most of these bacteria assume a spiral shape sometime during their life cycle [5,25] and they can also utilize a variety of electron donors and acceptors (noted above), these characteristics are not unique to this group [2,4-6,10]. Presently, there is no molecular or biochemical characteristic known that is unique to this group of bacteria. Within ε-proteobacteria, two main orders, *Campylobacterales *and *Nautiliales*, are presently recognized [8,10,12,24] The *Campylobacterales *is made up of three families, *Campylobacteraceae*, *Helicobacteraceae *and *Hydrogenimonaceae*, whereas the *Nautiliales *order is comprised of three genera (*Nautilia, Lebetimonas *and *Caminibacter*) [8,10,12,24]. Except for the 16S rRNA, very little sequence information is available for species belonging to the *Hydrogenimonaceae *family and the *Nautiliales *order.

In the past few years, genomic sequences of several ε-proteobacterial species from the *Campylobacterales *order have become available. The completely sequenced genomes include those from: *Helicobacter pylori *26695 [26], *H. pylori *J99[27], *H. hepaticus *ATCC 51449 [28], *Campylobacter jejuni *NCTC 11168 [29], *C. jejuni *RM1221 [30],*Wolinella succinogenes *DSM 1740 [23] and *Thiomicrospira denitrificans *ATCC 33889 [31]. In addition, genomes of several *Campylobacter *species (viz. *C. lari, C. coli, C. upsaliensis *and *C. fetus*) and *C. jejuni subsp. jejuni *strains (viz.*HB93-13, 84-25, CF93-6, 260.94, 11168 *and *81-176*) are now at assembly stage[30] and sequence information from them is available in the NCBI database. The availability of these sequences has opened new windows for discovering novel molecular characteristics that are unique to these bacteria and can be used for their diagnostics as well as for biochemical and functional studies. Earlier comparative genomic studies on ε-proteobacteria have examined a number of aspects of their gene/protein contents [14,23,26-30,32-34]. Of these, the studies by Eppinger et al. [14] and Fouts et al. [30] are particularly detailed. In these works, genes/proteins that are unique to individual genomes were identified as well as genes that are commonly shared by, but not uniquely present, in a number of these bacteria (viz. *H. pylori, H. hepaticus, C, jejuni and W. succinogenes*). Pair-wise comparison of the gene contents of these bacteria, functional classification of their genomic inventory, synteny and co-linearity of genes in various genomes, and examples of gene losses as well as recombination were also reported [14,30]. Additionally, Coenye and Vandamme [35] have carried out studies to identify genes that have been laterally transferred between ε-proteobacteria and other bacteria. However, thus far no comparative study has examined or identified genes/proteins that are uniquely found in ε-proteobacteria at different taxonomic levels. Such genes and proteins, because of their specificity, provide novel means for diagnostics and taxonomic studies [36-39] and for discovering important physiological characteristics that are unique to these bacteria.

In our recent work, we have used comparative genomics to identify a large number of signature proteins that are specific for either alpha proteobacteria [40], chlamydiae [38] or Actinobacteria [39]. In the present work, we have carried out systematic BLAST searches on all open reading frames (ORF) in the genome of *Wolinella succinogenes *DSM 1740 and *Campylobacter jejuni *RM1221 to identify whole genes/proteins (i.e. signature proteins) that are unique to ε-proteobacteria. These studies have led to identification of 49 genes/proteins that are uniquely present in various sequenced ε-proteobacteria (including *Thiomicrospira*), as well as many other proteins that are limited to certain subgroups within the *Campylobacterales *order. Additionally, we also describe a number of conserved indels in widely distributed proteins that are specific for either all-available ε-proteobacteria or for certain subgroups among them. The identified signature proteins and indels comprise rare genetic changes that have been introduced at various stages during the evolution of *Campylobacterales *(ε-proteobacteria) and their species distribution patterns are supported by the branching order of these species in phylogenetic trees.

## Results and discussion

These studies were undertaken to identify molecular characteristics that are uniquely shared by either all sequenced ε-proteobacteria species, or their subgroups, but which generally are not found in any other organism. Three different kinds of molecular signatures that are specific for ε-proteobacteria are described in the present work. The first of these consists of whole proteins or open reading frames (ORFs) that are uniquely found in ε-proteobacteria. The other two characteristics are comprised of rare genetic changes (RGCs) consisting of either conserved inserts or deletions (indels) in widely distributed proteins that are specific for the ε-proteobacterial homologs as well as a gene-fusion event within this group of bacteria. A brief description of these molecular signatures and their evolutionary significances are discussed below.

### Whole proteins or ORFs that are unique for the epsilon-proteobacteria (Campylobacterales order) and Helicobacteraceae family

The ε-proteobacteria-specific proteins were identified as described in the Methods section. Generally, a protein was considered to be epsilon-proteobacteria specific if all significant alignments (or hits) in a PSI-BLAST search with the query protein were from ε-proteobacteria species. In a few cases, where the E values of 1 or 2 hits from other species also exhibited borderline significance, but there was a large increase in E value from the last ε-proteobacteria hit in the search to these other proteins, such proteins were also regarded as ε-proteobacteria-specific. In Table 1, I list some characteristics of 53 proteins that could be regarded as specific for most sequenced ε-proteobacteria based on these criteria. Forty-one of these 53 proteins were present in all sequenced ε-proteobacteria genomes and for them all significant alignments/hits were from this group. However, in three instances (viz. WS0216, WS0260 and WS1495) the E value for one ε-proteobacteria was just above the default threshold value (.005) for significance. For three other proteins, WS0316, WS1874 and WS2146, 1–3 hits from other bacteria exhibited borderline significance, but there was a large jump in E values from the last ε-proteobacteria hit to these other proteins (see Table 1), indicating that these proteins are also ε-proteobacteria-specific. Eight other proteins in this Table (WS0865, WS1211, WS1235, WS1329, WS1640, WS1752, WS1771 and WS2059) are missing in 1–2 ε-proteobacteria species, which could be due to selective gene loss [33]. Of these 8 proteins, WS1211, WS1752 and WS2059 are present in almost all sequenced ε-proteobacteria except *T. denitrificans*. The phylogenetic position of *T. denitrificans *within ε-proteobacteria is presently not clear (discussed later). Hence, absence of these proteins in *T. denitrificans *could be explained by either earlier divergence of this species in comparison to other sequenced *ε*-proteobacteria, or due to gene loss.

For the protein WS0230 listed in Table 1, in addition to various ε-proteobacteria, homologs with very low E values (e-90 range) were also found in two δ-proteobacteria belonging to the *Desulfovibrio *genus. In phylogenetic trees based on 16S rRNA [2,41], various proteins [42,43], and in analyses based on conserved indels [44], δ-proteobacteria generally branch in close proximity to the ε-proteobacteria. Hence, the shared presence of the WS0230 homologs in *Desulfovibrio *genus and ε-proteobacteria may reflect either a deep phylogenetic relationship that exist between these two groups [43-45], or it could result from lateral gene transfer [46]. Based on the available data we are unable to distinguish between these possibilities. However, it is interesting to note that a 1 aa insert in a conserved region of the RecA protein, which was previously indicated to be specific for ε-proteobacteria [44], and is present in all available ε-proteobacteria homologs, is also commonly present in *Desulfovibrio *and *Lawsonia *species (belonging to *Desulfovibrionaceae *family) (results not shown).

Table 1 also lists the available information regarding possible cellular functions of these proteins. Most of these proteins are of unknown functions. However, in a number of cases weak but significant similarity is observed to conserved domains found in other proteins in the databases [47], or to particular COG families [48]. The information of this kind, along with the genomic context of these ORFs, provide useful leads for exploring the cellular functions of these conserved hypothetical proteins [49-52]. Of the proteins that are found in all sequenced ε-proteobacteria, WS0266 and WS0802 were experimentally identified as plasminogen binding proteins [53]. It has been suggested that these proteins may enable these bacteria to coat their exterior surface with plasminogen and thus they could be involved in enhancing their virulence. The putative functions of several other proteins are indicated in Table 1 and they include a putative helicase (WS0086), a Cbb3 type cytochrome oxidase (WS0180), a protein related to the FixH family (WS0185) of *Rhizobium*, a protein WS0316 containing the RDD domain, two proteins (WS0476 and WS0480) which contain molybdopterin_binding (MopB) domain found in NADH oxidoreductase I. Also found were two proteins implicated in flagellar function (WS0490 and WS0575) [23], a protein (WS0520) with TonB domain and another protein (WS1874) containing a domain related to the DNA polymerase delta subunit, a protein (WS2146) showing some similarity to Sua5 domain involved in binding to double stranded DNA, and a protein WS0230 showing similarity to deacylase domain. In addition, several proteins are predicted to be either periplasmic or membrane proteins. It should be emphasized that most of these functional predictions or annotations are based on weak similarity to conserved domains (CD) as identified by the CD search program implemented with the BLAST program [47]. Although this information is very useful, the actual functions of most of these proteins, which exhibit very little similarity to other molecules in the database, remain to be determined. Among the proteins listed in Table 1 that are missing in some ε-proteobacteria, WS1211 is a homolog of the *C. jejuni *invasion antigen (CiaB), which is recognized as an important factor in its pathogenicity[14,54]. Of the proteins listed in Table 1, 10 proteins (WS133-WS134, WS184-WS185, WS447-WS448, WS1039-WS1040 and WS1495-WS1496) are present in clusters of two in the genome, and they could be involved in related functions [51,52].

**Figure 1:**
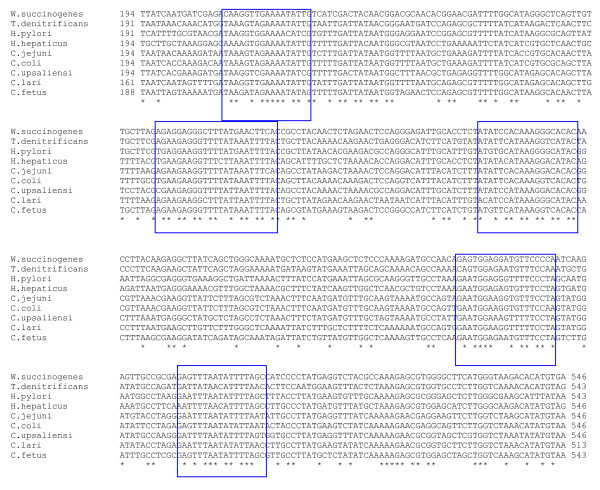
Partial nucleotide sequence alignment for an ε-proteobacterial specific protein WS0086. The initial part of this alignment, which is less conserved and some of which is also missing in *C. lari*, is not shown. The asterisks (*) denote residues that are completely conserved. A number of conserved regions that are suitable for designing PCR primers or other diagnostic probes are boxed.

Several of the proteins listed in Table 1 (e.g., WS0086 and WS2123) exhibit a high degree of sequence conservation across various ε-proteobacteria species. A partial nucleotide sequence alignment for the WS0086 coding sequence for various ε-proteobacterial species is shown in Figure 1. A large number of positions in the alignments are completely conserved in various *Campylobacterales *species and there are several long stretches (boxed) showing a high degree of sequence conservation. The PCR primers and other molecular probes based on these conserved regions could provide novel and specific means for identification of both new, as well existing *Campylobacterales *species and possibly different ε-proteobacteria.

The comparative analysis of *W. succinogenes *genome has also identified 11 proteins that are uniquely found in *Wolinella *and *Helicobacter *species (Table 2). Of these 11 proteins, the first 7 are present in all 4 of the sequenced species/strains from these genera, whereas the last 4 proteins are only found in *W. succinogenes *and *H. hepaticus *but missing in the two *H. pylori *strains. All of these proteins are of unknown function. The *Wolinella *and *Helicobacter *genera are part of the *Helicobacteraceae *family and these uniquely shared proteins provide potential molecular markers for this family.

Our analysis also reveals that 99 proteins in the genome of *W. succinogenes *DSM 1740 show no significant similarity to any other protein in the databases [see Additional file 1]. Barr et al. [23] have previously indicated a much higher number (i.e. 490) of such proteins. However, since their analysis, genomes of several ε-proteobacteria as well as numerous other organisms have become available [28,30,31,55]. Because of this, and our employment of more stringent criteria for identification of group-specific proteins, the number of such proteins is considerably smaller than indicated originally [23]. Sixteen of these proteins are present in seven clusters (WS0261-WS0262; WS0531-WS0532; WS1446-WS1447; WS1573-WS1674; WS1888-WS1889; WS2027-WS2028-WS2029; WS2032-WS2033-WS2034) in the *W. succinogenes *DSM 1740 genome.

### Proteins specific for the Campylobacter genus

We have also performed BLAST searches on various proteins found in the genome of *C. jejuni *RM1221 to identify proteins that are unique to the *Campylobacter *species. Fouts et al. [30], who sequenced the genomes of several *Campylobacter *species/strains had reported comparative studies on them. Their work identified several proteins that were specific for the *C. jejuni *RM1221 and *C. jejuni *NCTC 11168 strains (Supplementary Table S7 in their paper), but they did not look for proteins that were uniquely shared by either all or different Campylobacter species. Our analyses have identified 15 proteins (Table 3) that are uniquely present in all of the sequenced *Campylobacter *species viz. *C. fetus*, *C. lari, C. upsaliensis, C. coli *and *C. jejuni *(NCTC 11168, RM1221, HB93-13, 84-25, CF93-6, 260.94, 11168 and 81-176). Three additional proteins listed in Table 3, CJE0368, CJE1499 and CJE1574 are missing in only one of the *Campylobacter *species, which is likely due to gene loss. Eighteen other proteins (Table 4) are present in all of the *Campylobacter *species, except *C. fetus*. Among the sequenced *Campylobacter *species, *C. fetus *exhibits deepest branching in various phylogenetic trees (see next section). Hence, the absence of these proteins in *C. fetus *could be explained by their introduction in a common ancestor of the other *Campylobacter *species after branching of *C. fetus*. Ten other proteins (Table 5) are commonly present in *C. upsaliensis, C. coli *and *C. jejuni *only indicating a closer relationship among these species. The genes for these proteins were likely introduced or evolved in a common ancestor of these three species. Likewise, 28 other proteins listed in Table 6, which are only found in *C. coli *and *C. jejuni *(different strains) points to a specific relationship between these species to the exclusion of all others. Most of these proteins are of unknown function. However, in a few cases, where any similarity to conserved domain present in other proteins has been identified by BLAST searches, such information is noted in various Tables.

These analyses have also identified a large number of proteins that are specific for the *C. jejuni *species (Table 7). The first 5 proteins listed in this table are present in all sequenced *C. jejuni *strains (NCTC 11168, RM1221, HB93-13, 84-25, CF93-6, 260.94, 11168 and 81-176), whereas the remainder are missing or have been lost from a few of the strains.

### Conserved indels and other rare genetic changes specific for epsilon proteobacteria

Conserved indels in protein sequences provide another useful kind of molecular signatures for taxonomic and diagnostic studies. In our recent work, conserved indels that are distinctive characteristics of many different groups of bacteria (e.g., Chlamydiae, Proteobacteria, alpha proteobacteria, *Actinobacteria, Cyanobacteria, Deinococcus-Thermus, Aquificae*, etc.) have been identified [44,56-60]. To identify conserved indels that may be specific for ε-proteobacteria, the sequence alignments of various proteins constructed in earlier work were examined. These studies have led to identification of 4 conserved indels that are specific for this group. The characteristics of these indels and of the proteins in which they are found are briefly described below.

**Figure 2:**
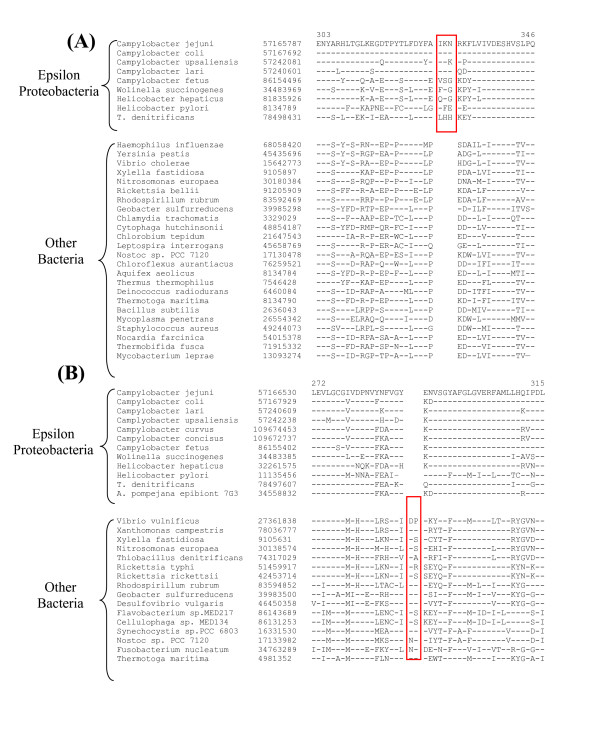
Partial sequence alignments of the B protein from exinuclease ABC complex (A) and phenylalanyl-tRNA synthetase (B) showing two conserved indels that are specific for ε-Proteobacteria and not found in other organisms. The dashes (-) in the alignment show identity with the amino acid on the top line. The accession numbers of the sequences (second column) and position of the sequence in *C. jejuni *homolog (on top) are indicated. Sequence information for only representative species is shown.

**Figure 3:**
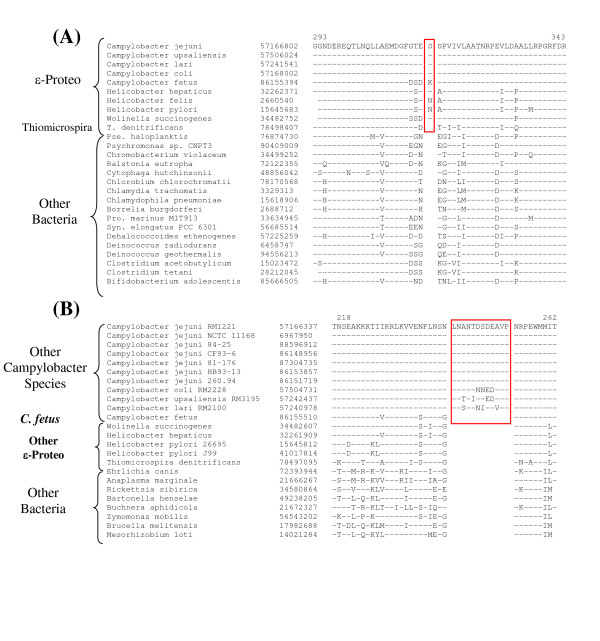
Partial sequence alignments of the FtsH protease (A) and RNA polymerase β' subunit (B) showing two conserved indels that are specific for the indicated subgroups of ε-Proteobacteria. The dashes (-) denote identity with the amino acid on the top line. Sequence information for only representative species is shown.

In Figure 2, I present sequence information for two conserved indels that are uniquely present in various sequenced ε-proteobacterial homologs, but which are not found in the corresponding proteins from any other organism. The first of these indels is a 3 aa insert in the B protein of the Uvr ABC system (Fig. 2A), which plays a key role in the nucleotide excision repair process [61]. The second indel consists of a 2 aa deletion in the enzyme phenylalanyl-tRNA synthetase (Fig. 2B), which is required for protein synthesis. Both these proteins are widely distributed in bacteria and sequence information for only representative species from other bacteria is presented. The indels in both these proteins are flanked by highly conserved regions and the unique presence of these indels in all available ε-proteobacteria homologs strongly indicate that they are distinctive molecular characteristics of these bacteria. Two additional conserved indels that are specific for only certain ε-proteobacteria are shown in Figure 3. The top panel in this Figure shows a 1 aa insert in the FtsH protease that is uniquely present in all sequenced ε-proteobacteria, except *T. denitrificans*. The absence of this indel in various other bacteria as well *T. denitrificans *indicates that this indel is an insert that was introduced in a common ancestor of *Helicobacter, Campylobacter *and *Wolinella*, after the branching of *T. denitrificans*. The lower panel in Fig. 3 shows a highly conserved insert in the β '-subunit of RNA polymerase (RpoC) that is uniquely present in various *Campylobacter *species, except *C. fetus*. RpoC homologs are present in all sequenced genomes and the identified insert is not found in any other ε-proteobacteria or other organism. This insert was likely introduced in a common ancestor of the *Campylobacter *after branching of *C. fetus*.

**Figure 4:**
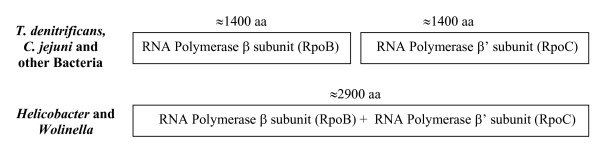
Diagrammatic representation of the arrangements of two largest subunits of RNA polymerase, i.e. β subunit (RpoB) and β' subunit (RpoC) in different bacteria. In contrast to other bacteria where these proteins are made as distinct polypeptides, in *Helicobacter *and *Wolinella *a rare genetic event has led to fusion/joining of the genes for these proteins so that they are now made as a single large polypeptide.

In addition to these conserved indels, Zakaharova et al. [62] have identified a rare genetic event that causes fusion of two different genes within certain groups of ε-proteobacteria. The two largest and highly conserved subunits of RNA polymerase (RpoB and RpoC, each approximately 1400 aa) are encoded by two distinct genes in various bacteria [62]. However, a rare genetic event has led to the fusion of these genes in *Helicobacter *and *Wolinella *species, such that RpoB and RpoC are now made as a single large polypeptide (≈ 2900 aa) (Fig. 4). In contrast, in *Campylobacte*r and *T. denitrificans*, similar to other bacteria, separate genes encode for these proteins. This rare genetic event provides evidence of a specific relationship between *Helicobacter *and *Wolinella *species, which are part of the *Helicobacteraceae *family.

### Evolutionary significance of the signature proteins and conserved indels

**Figure 5:**
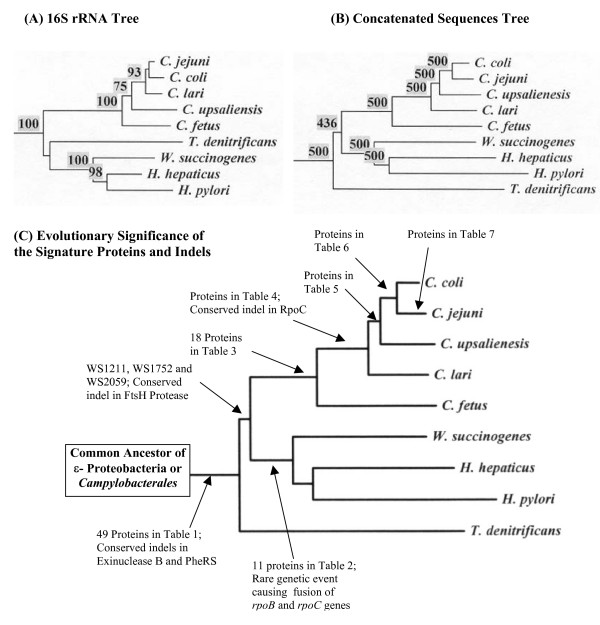
Phylogenetic trees based on (A) 16S rRNA and (B) concatenated sequences for 9 proteins (AlaRS, Gyrase A, Gyrase B, EF-Tu, EF-G, Hsp60, Hsp70, RpoB and RpoC) containing 7919 aligned positions. The sequences were bootstrapped either 100 (A) or 500 times (B) and bootstrap scores for all nodes above 50% are shown. (C) A model depicting the evolutionary stages where different *Campylobacterales*- (or ε-proteobacteria) specific proteins and other RGCs were introduced.

It is important to understand at what point during the evolution of ε-proteobacteria, the above-described molecular characteristics evolved or were introduced. To determine their evolutionary significance, phylogenetic trees were constructed for the sequenced ε-proteobacteria species based on 16S rRNA and a concatenated dataset of sequences for 9 highly conserved proteins (viz. RpoB, RpoC, Hsp70, Hsp60, elongation factor (EF)-Tu, EF-G, Gyrase A, Gyrase B and alanyl-tRNA synthetase). In the 16S rRNA tree, the ε-proteobacterial species under consideration formed two clades (Fig. 5A). One clade consisted of various *Campylobacter *species whereas the other clade included *Helicobacter*, *Wolinella *and *T. denitrificans*. In the latter clade, *T. denitrificans *formed a deep branching outgroup of the *Helicobacter *and *Wolinella *species, but a specific association of *T. denitrificans *to these species was not supported by the bootstrap score of the node (<50%) (Fig. 5A) [8,12]. In contrast to the rRNA tree, in the tree based on concatenated protein sequences, all of the internal nodes were reliably resolved. In this tree, *T. denitrificans *formed a deep branching lineage showing no specific relationship to either the *Helicobacter/Wolinella *clade or to the *Campylobacter *species (Fig. 5B). A similar deep branching of *T. denitrificans *in comparison to other sequenced ε-proteobacteria is observed in phylogenetic trees based on Hsp70, RpoC, Gyrase A, Gyrase B and EF-Tu protein sequences (results not shown).

Using the above trees as reference points, the evolutionary stages where different ε-proteobacteria-specific genes/proteins or other molecular signatures likely evolved is depicted in Fig. 5C. The genes for the first 49 proteins listed in Table 1 as well as the conserved indels in PheRS and exinuclease B protein, which are unique to almost all sequenced ε-proteobacteria, were likely introduced in a common ancestor of the *Campylobacterales *or ε-proteobacteria. The genes for the last three proteins listed in Table 1 (viz. WS1211, WS1752 and WS2059) that are absent in *T. denitrificans *but present in all (or most) other ε-proteobacteria were likely introduced in a common ancestor of the *Helicobacter, Wolinella *and *Campylobacter *after the divergence of *T. denitrificans*. The insert in the FtsH protease was also likely introduced at this stage. The proteins listed in Table 2 were introduced in a common ancestor of the *Wolinella *and *Helicobacter *genera, and it is expected that some of them will constitute distinctive characteristics of the *Helicobacteraceae *family. The rare genetic event leading to the fusion of *rpoB *and *rpoC *genes also occurred at a similar stage. The proteins listed in Tables 3 to 7 that are unique to either all sequenced *Campylobacter *species or various species within this genus, were introduced at different stages in the evolution of this group (Fig. 5C). The observed species distribution patterns of these proteins strongly support the branching pattern of *Campylobacter *species in the phylogenetic trees (Figs. 5A and 5B). The inference from these proteins and the phylogenetic trees that *C. fetus *is one of the deepest branching species within the *Campylobacter *genus is also strongly supported by the large insert in RpoC (Fig. 3B), which is present in all *Campylobacter *species except *C. fetus*.

## Conclusion

The comparative genomics of ε-proteobacteria reported here have led to identification of a large number of molecular signatures (e.g., whole proteins, conserved indels and a gene-fusion event) that are distinctive characteristics of these bacteria. Our analyses indicate that these characteristics have been introduced at various stages in the evolution of ε-proteobacteria, but once introduced, they were generally stably retained in various descendents of these lineages with minimal gene loss or lateral gene transfer to other bacteria. Sequence information for these proteins or molecular signatures is presently available only from the *Campylobacterales *species and no information is available from the *Nautiliales *order, which comprise the other main group within ε-proteobacteria. However, the genomes of several ε-proteobacteria (e.g. *Nautilia, Caminibacter, Arcobacter, Sulfurovum, Nitratiruptor*) covering all of its main groups are currently in progress (noted in ref. [10]). Based upon our work on signature sequences for other groups of bacteria [56-59], we expect that many of the signatures identified in the present work (Table 1) will also be found in different ε-proteobacteria, whereas several other will prove to be specific for only the *Campylobacterales *order. The primary sequences of many of these genes/proteins are highly conserved and they provide novel diagnostic tools for these bacteria by means of PCR amplification and fluorescence *in situ *hybridization methods. Monoclonal and polyclonal antibodies based upon these proteins provide another means for their detection. Additionally, these *Campylobacterales *or ε-proteobacteria specific proteins also provide potential targets for developing therapeutics and vaccines that are specific for these bacteria. The identified signature proteins and RGCs also provide novel and definitive molecular means for circumscribing a number of taxonomic groups within *Campylobacterales *(ε-proteobacteria) and for identifying species belonging to these groups.

The cellular functions of most of the ε-proteobacteria-specific proteins are not known. Although a number of these proteins exhibit weak sequence similarity to conserved domains in other proteins, their actual functions may be quite different, and determining them constitute an important task for the future. Likewise, it is also of much interest to understand the functional significance of the conserved indels in various proteins (viz. RpoC, PheRS, FtsH, exinuclease B) that are specific for different taxonomic groups/clades of ε-proteobacteria. Since these indels, which are located in highly conserved regions, are retained by all (available) members of these clades it is highly likely that they are functionally important (and essential) for these bacteria. Thus, it is of much importance to understand how the functions of these proteins are modified by these indels and the physiological significance of these modifications for these bacteria. Further studies on these ε-proteobacteria specific proteins and indels thus may lead to the discovery of novel biochemical and physiological characteristics that are uniquely shared by these bacteria.

## Methods

### Identification of proteins that are specific for epsilon proteobacteria

To identify proteins that are specific for ε-proteobacteria, all proteins in the genomes of *W. succinogenes *DSM 1740 [23] were analyzed. This genome was chosen for a number of reasons. First, of the sequenced ε-proteobacteria genomes, *W. succinogenes *genome is among the largest (2.11 Mb) with 2043 ORFs [23]. Hence, one expects that minimal gene loss has occurred in this bacterium and that it should contain maximal number of genes that may be present in other ε-proteobacteria. Second, phylogenetic and comparative studies have indicated that *W. succinogenes *forms an outgroup to various *Helicobacter *species and thus lies in an intermediate position between members of the *Helicobacteraceae *and *Campylobacteraceae *families [6,14]. Thus, BLAST searches on proteins from this genome should enable us to identify proteins that are unique to the *Helicobacteraceae *family as well as those shared with other taxonomic groups of ε-proteobacteria. To identify proteins that are specific for the *Campylobacter *species, the genome of *C. jejuni *RM1221 was analyzed. The BLASTp searches were initially performed on each individual protein or ORF in these genomes against all available sequences in the NCBI sequence database, to identify all related gene/protein in other organisms [63,64]. These searches were performed using the default parameters as set by the BLAST program, except that the low complexity filter was turned off. The expected values (E-values) of different hits from these searches were inspected to identify putative ε-proteobacteria-specific proteins [38,40]. The proteins that were of interest to us generally involved large increase in E-values from the last ε-proteobacteria hit in the blast search to the first hit from any other organism. Further, the E values of these latter hits were expected to be in a range higher than 10^-4^, which indicates weak level of similarity that could occur by chance. However, higher E-values are sometimes acceptable for smaller proteins as the magnitude of the E-value depends upon the length of the query sequence [63]. All promising proteins identified by the above criteria were further analyzed using the position-specific iterated (PSI) BLAST program [63]. This program creates a position-specific scoring matrix from statistically significant alignments produced by the BLASTp program and then searches the database using this matrix. The PSI-BLAST program is more sensitive in identifying weak but biologically relevant sequence similarity as compared to the BLASTp program [63]. The output of the PSI-BLAST program divides the various hits into two categories, i.e. sequences producing significant alignment versus those where the E values are worse than the threshold (default value set at .005). For most of the proteins that are indicated to be specific for different subgroups within ε-proteobacteria, all significant alignments were from the indicated groups. In a few cases, where an isolated hit has an E value slightly below the threshold value (arbitrarily set), but there was a large jump in E value from the last ε-proteobacteria hit, such proteins were also regarded as specific for the indicated groups. All of the identified group-specific proteins were also examined for the presence of any conserved domain [47] and this information along with the genome identification number of the protein, its accession number, sequence length, etc. was tabulated. In the description of various proteins in the text, the "WS" and "CJE" parts of the descriptors indicate the identification numbers of the proteins in the genomes of *W. succinogenes *DMS 1740 and *C. jejuni *RM1221, respectively.

### Identification of conserved indels that are specific for epsilon proteobacteria

Multiple sequence alignments for large number of proteins have been created in our earlier work [44,56,60]. To search for conserved indels that might be specific for ε-proteobacteria, these alignments were visually inspected to identify any indel that was uniquely present in ε-proteobacteria species, and which was flanked by conserved sequences. The indels that were not flanked by conserved regions were not considered. The specificity of these indels for ε-proteobacteria was evaluated by carrying out detailed BLAST searches on short sequence segments (usually between 60–100 aa) containing the indel and the flanking conserved regions. The purpose of these BLAST searches was to obtain sequence information from all available bacteria homologs to determine the presence of the identified indels in various species. The sequence information for these indels was compiled into signature files such as those presented in Figures 2 and 3.

### Phylogenetic analysis

Phylogenetic trees for the sequenced ε-proteobacteria species were constructed based on 16S rRNA sequences as well as a number of conserved proteins (viz. RNA polymerase β subunit (RpoB), RNA polymerase β ' subunit (RpoC), DNA gyrase A subunit (GyrA), DNA gyrase B subunit (GyrB), Hsp70, Hsp60, alanyl tRNA synthetase (AlaRS), elongation factor-G (EF-G) and elongation factor-Tu (EF-Tu) proteins) The 16S rRNA and protein sequences were downloaded from the Ribosomal Database Project-II site [65] and NCBI databases, respectively and aligned using the CLUSTALx program [66]. A neighbor-joining bootstrapped trees based on rRNA sequences was constructed by the Juke's and Cantor [67] method. The sequences for various proteins were concatenated into a large dataset containing 7919 aligned positions (RpoB (1440), RpoC (1559), GyrA (880), GyrB (814), Hsp70 (661), Hsp60 (552), AlaRS (912), EF-G (698) and EF-Tu (403)) and a neighbor-joining bootstrap tree based on this was constructed by Kimura's methods [68]. All gaps in the sequences were omitted during phylogenetic analyses. The trees were constructed using the PHYLIP [69] and the TREECON programs [70] and they were rooted using the chlamydiae species which is a deep branching group in comparison to ε-proteobacteria [41-43,45].

## Abbreviations

CD, conserved domain; Indel, insert or deletion; ORF, open reading frame; ORFans, open reading frames of unknown functions; PheRS, phenylalanyl-tRNA synthetase; RGC, rare genetic change; RpoB and RpoC, RNA polymerase β and β '-subunits, respectively.

## Supplementary Material

Additional file 1 Proteins that are Unique to *Wolinella succinogenes *DSM 1740. In BLASTp and PSI-BLAST searches, no significant similarity to these proteins was detected for any other protein. The identification numbers of these proteins in *Wolinella succinogenes *DMS 1740 genome, accession numbers, protein lengths and information regarding putative function, if known, are provided. Click here for file

## Figures and Tables

**Table 1:** Proteins that are uniquely present in most epsilon proteobacteria (*Campylobacterales*)

**Wolinella Genome ID No.**	**Accession Number**	*Length*	Possible/Predicted Function	Comments
WS0030	NP_906303.1	68 aa	Probable periplasmic protein, tat-domain	All significant hits from ε-Proteobacteria
WS0086	NP_906354	181 aa	Putative helicase, gnl|CDD|14084, COG4951	All significant hits from ε-Proteobacteria
WS0133	NP_906397	397 aa	Putative integral membrane protein	All significant hits from ε-Proteobacteria
WS0134	NP_906398.1	214 aa	Conserved hypothetical protein	All significant hits from ε-Proteobacteria
WS0154	NP_906417.1	336 aa	Probable membrane protein	All significant hits from ε-Proteobacteria
WS0159	NP_906422	203 aa	Conserved hypothetical protein, unknown function	All significant hits from ε-Proteobacteria
WS0169	NP_906432	92 aa	Possible membrane protein (corresponds to Cj0692c and HP0748)	All significant hits from ε-Proteobacteria
WS0172	NP_906435	675 aa	Putative membrane protein, similar to HP0358	All significant hits from ε-Proteobacteria
WS0180	NP_906442	74 aa	Related to Cbb3-type cytochrome oxidase, subunit 3, gnl|CDD|13876, COG4736	All significant hits from ε-Proteobacteria
WS0184	NP_906445.1	205 aa	Probable membrane protein	All significant hits from ε-Proteobacteria
WS0185	NP_906446	163 aa	Related to FixH protein, gnl|CDD|23975	All significant hits from ε-Proteobacteria
WS0216	NP_906474	330 aa	Conserved hypothetical protein, unknown function	All significant hits from ε-Proteo; E value for *C. upsaliensis *is higher than the threshold.
WS0260	NP_906515.1	142 aa	Conserved hypothetical protein, unknown function	All significant hits from ε-Proteobacteria; E value for *C. lari *is above the threshold.
WS0266	NP_906520	271 aa	Conserved protein, *H. pylori *homolog may be related to the plasminogen binding protein pgbA.	All significant hits from ε-Proteobacteria; Jonsson et al. (2004)
WS0316	NP_906567	163 aa	Conserved protein related to the RDD family; gnl|CDD|25144, pfam06271	Besides ε-Proteo, three other hits were below the threshold value; Large jump in E value from last ε-Proteo (6E-15) to the first of these hits (.003).
WS0447	NP_906689	328 aa	Putative membrane protein, corresponds to antigen P44Hh9 of *H. hepaticus*.	All significant hits from ε-Proteobacteria
WS0448	NP_906690	276 aa	Probable periplasmic protein	All significant hits from ε-Proteobacteria
WS0476	NP_906716	77 aa	NuoE, Putative NADH Oxidoreductase I	All significant hits from ε-Proteobacteria
WS0480	NP_906720	428 aa	Putative NADH Oxidoreductase I	All significant hits from ε-Proteobacteria
WS0490	NP_906728	778 aa	Flagellar functional protein, Pf1a	All significant hits from ε-Proteobacteria
WS0520	NP_906757.1	247 aa	TonB domain protein	All significant hits from ε-Proteobacteria
WS0563	NP_906797	164 aa	Putative integral membrane protein; identified by similarity to PIR:B71953	All significant hits from ε-Proteobacteria
WS0575	NP_906809	217 aa	Putative lipoprotein, The *C. lari *homolog is a secreted protein involved in flagellar motility.	All significant hits from ε-Proteobacteria.
WS0604	NP_906835	390 aa	Probable periplasmic protein	All significant hits from ε-Proteobacteria
WS0802	NP_907015	333 aa	Probable lipoprotein; identified as plasminogen binding protein pgpB.	All significant hits from ε-Proteobacteria; Jonsson et al. (2004)
WS0865	NP_907074.1	126 aa	Conserved hypothetical protein, unknown function	All significant hits from ε-Proteobacteria; missing in *C. jejuni*.
WS1039	NP_907239	156 aa	Conserved hypothetical protein, unknown function	All significant hits from ε-Proteobacteria
WS1040	NP_907240.1	236 aa	Conserved hypothetical protein, unknown function	All significant hits from ε-Proteobacteria
WS1235	NP_907415	412 aa	Putative periplasmic protein; COG5659	All significant hits from ε-Proteobacteria; not found in *H. hepaticus*.
WS1244	NP_907424	167 aa	Putative lipoprotein	All significant hits from ε-Proteobacteria
WS1329	NP_907504	246 aa	Putative periplasmic protein	All significant hits from ε-Proteobacteria; absent in *H. pylori*.
WS1344	NP_907515.1	123 aa	Putative periplasmic protein	All significant hits from ε-Proteobacteria
WS1349	NP_907520.1	110 aa	Probable membrane protein	All significant hits from ε-Proteobacteria
WS1485	NP_907639.1	89 aa	Probable integral membrane protein	All significant hits from ε-Proteobacteria
WS1495	NP_907647	87 aa	Conserved hypothetical protein, unknown function	All significant hits from ε-Proteo; The E value for *C. lari *(next best hit) above the threshold.
WS1496	NP_907648	208 aa	Probable periplasmic protein	All significant hits from ε-Proteobacteria
WS1640	NP_907771	117 aa	Probable integral membrane protein	All significant hits from ε-Proteobacteria; absent in *H. pylori*.
WS1730	NP_907855	183 aa	Conserved hypothetical protein, unknown function	All significant hits from ε-Proteobacteria
WS1755	NP_907877	168 aa	Probable lipoprotein	All significant hits from ε-Proteobacteria
WS1771	NP_907893	183 aa	Putative membrane protein	All significant hits from ε-Proteobacteria; absent in *H. pylori*.
WS1773	NP_907895	351 aa	Putative membrane protein	All significant hits from ε-Proteobacteria
WS1777	NP_907899.1	80 aa	Conserved hypothetical protein, unknown function	All significant hits from ε-Proteobacteria
WS1814	NP_907930.1	85 aa	Conserved hypothetical protein, unknown function	All significant hits from ε-Proteobacteria
WS1874	NP_907984	352 aa	HolA, DNA polymerase III, delta subunit; gnl|CDD|11180, COG1466	All significant hits except one from ε-Proteo; E value for *Geo. metallireducens *hit (.003).
WS1965	NP_908068	121 aa	Conserved hypothetical protein, unknown function	All significant hits from ε-Proteobacteria
WS1990	NP_908093	118 aa	Conserved domain DUF 177: COG1399	All significant hits from ε-Proteobacteria
WS2120	NP_908218.1	162 aa	Conserved hypothetical protein, unknown function	All significant hits from ε-Proteobacteria
WS2123	NP_908221	246 aa	Conserved hypothetical protein, unknown function	All significant hits from ε-Proteobacteria
WS2146	NP_908240	147 aa	Contains Sua5_yciO_yrdC domain involved in binding to dsRNA; gnl|CDD|15330	All significant hits except one from ε-Proteo; E value changes from 3e-19 to 2e-4 for *M. synoviae*
WS0230	NP_906487	432 aa	Show significant similarity to deacylase domain; gnl|CDD|12932, COG3608	Besides ε-Proteo, homologs with very low E values also present in two *Desulfovibrio *species.
WS2059	NP_908159	259 aa	Conserved hypothetical protein, unknown function	All significant hits from ε-Proteo; not found in *T. denitrificans*.
WS1752	NP_907874	145 aa	Conserved hypothetical protein, unknown function	All significant hits from ε-Proteo; not found in *C. fetus *and *T. denitrificans*.
WS1211	NP_907393	621 aa	Homologous to CiaB invasion antigen of *C. jejuni*	All significant hits from ε-Proteo; not found in *H. pylori *and *T. denitrificans*. Konkel et al. (1999)

The species distribution of these proteins was determined by BLASTp and PSI-BLAST searches as described in the Methods section. Unless otherwise indicated all of these proteins are uniquely found in the following sequenced genomes: *H. pylori *26695, *H. pylori *J99, *H. hepaticus *ATCC 51449, *C. jejuni *(various strains: NCTC 11168, RM1221, *HB93-13, 84-25, CF93-6, 260.94, 11168 *and *81-*176), *C. lari, C. coli, C. upsaliensis, C. fetus*, W. *succinogenes *DSM 1740 and *Thiomicrospira denitrificans *ATCC 33889.

**Table 2:** Proteins specific for the Wolinella and *Helicobacter *species (*Helicobacteraceae *family)

**Wolinella Genome ID No.**	**Accession Number**	*Length*	Possible/Predicted Function
WS0068	NP_906337.1	79	Hypothetical protein, unknown function
WS0584	NP_906816.1	171	Hypothetical protein, unknown function
WS1041	NP_907241.1	277	Hypothetical protein, unknown function
WS1051	NP_907250.1	80	Hypothetical protein, unknown function
WS1084	NP_907280.1	81	Hypothetical protein, unknown function
WS2139	NP_908234.1	161	Hypothetical protein, unknown function
WS2156	NP_908250.1	137	Hypothetical protein, unknown function
WS0682^a^	NP_906910.1	217	Hypothetical protein, unknown function
WS0805^a^	NP_907018.1	110	Hypothetical protein, unknown function
WS0828^a^	NP_907039.1	125	Hypothetical protein, unknown function
WS1624^a^	NP_907756.1	221	Hypothetical protein, unknown function

Homologs showing significant similarities to these proteins are only detected in the sequenced *Wolinella *and *Helicobacter *genomes (*W. succinogenes *DSM 1740, *H. pylori *26695, *H. pylori *J99 and *H. hepaticus *ATCC 51449).

^a ^These proteins are only found in *W. succinogenes *and *H. hepaticus*.

**Table 3:** Proteins specific for all sequenced *Campylobacter *species

**Geneomic ID [Accession Number]**	**Possible Function (length)**	**Geneomic ID [Accession Number]**	**Possible Function (length)**
CJE0368 [YP_178387]^b^	hypothetical protein (398)	CJE1156 [YP_179147]	putative membrane protein (154)
CJE0399 [YP_178418]	hypothetical protein (140)	CJE1173 [YP_179164]	outer membrane protein MapA (214)
CJE0627 [YP_178642]	probable membrane protein (144)	CJE1222 [YP_179210]	probable periplasmic protein (150)
CJE0751 [YP_178762]	hypothetical protein (144)	CJE1367 [YP_179354]	hypothetical protein (110)
CJE0754 [YP_178765]	membrane protein, putative (164)	CJE1499 [YP_179485]^b^	acyl carrier protein, putative (74)
CJE0790 [YP_178795]	membrane protein, putative (163)	CJE1574 [YP_179557]^a^	hypothetical protein (231)
CJE0888 [YP_178890]	prevent-host-death family protein (71)	CJE1623 [YP_179604]	putative ATP/GTP-binding protein (187)
CJE0986 [YP_178984]	Putative periplasmic protein (156)	CJE1670 [YP_179651]	hypothetical protein (142)
CJE1022 [YP_179020]	putative periplasmic protein (244)	CJE1745 [YP_179718]	hypothetical protein (230)

These proteins are uniquely found in all of the following *Campylobacter *genomes, unless otherwise indicated: *C. jejuni *(various strains: NCTC 11168, RM1221, *HB93-13, 84-25, CF93-6, 260.94, 11168 *and *81-176*), *C. lari, C. coli, C. upsaliensis *and *C. fetus*.

^a ^– missing in *C. upsaliensis*

^b ^– missing in *C. lari*.

**Table 4:** *Campylobacter*-specific proteins that are missing in *C. fetus*

Geneomic ID [Accession Number]	Possible Function (length)	Geneomic ID [Accession Number]	Possible Function (length)
CJE0037 [YP_178064]	hypothetical protein (215)	CJE0959 [YP_178957]	hypothetical protein (210)
CJE0039 [YP_178066]	hypothetical protein (107)	CJE1180 [YP_179170]^a^	hypothetical protein (83)
CJE0193 [YP_178217]	hypothetical protein (115)	CJE1221 [YP_179209]	prepilin-type N-terminal cleavage/methylation domain protein (220)
CJE0455 [YP_178474]	putative lipoprotein (299)	CJE1327 [YP_179314]	putative periplasmic protein (268)
CJE0470 [YP_178489]	membrane protein, putative (318)	CJE1351 [YP_179338]	hypothetical protein (67)
CJE0476 [YP_178495]	hypothetical protein (111)	CJE1378 [YP_179365]	hypothetical protein (106)
CJE0867 [YP_178869]	putative periplasmic protein (339)	CJE1572 [YP_179555]	lipoprotein, putative (176)
CJE0899 [YP_178901]	small hydrophobic protein (101)	CJE1849 [YP_179819]	probable periplasmic protein (254)
CJE0929 [YP_178931]^b^	putative lipoprotein (161)	CJE1890 [YP_179860]	Ribbon-helix-helix protein, copG family (82)

These proteins are uniquely present in all of these species unless otherwise noted: *C. jejuni *(various strains: NCTC 11168, RM1221, *HB93-13, 84-25, CF93-6, 260.94, 11168 *and *81-176*), *C. lari, C. coli*, and *C. upsaliensis*.

^a ^– missing in *C. upsaliensis*

^b ^– missing in some *C. jejuni *strains

**Table 5:** Proteins uniquely found in *C. jejuni, C. coli *and *C. upsalienesis*

Geneomic ID [Accession Number]	Possible Function (length)	Geneomic ID [Accession Number]	Possible Function (length)
CJE0052 [YP_178077]^a^	hypothetical protein (90)	CJE1095 [YP_179088]^a^	site-specific recombinase XerC, putative (86)
CJE0053 [YP_178078]^a^	hypothetical protein (67)	CJE1096 [YP_179089]^a^	hypothetical protein (76)
CJE0079 [YP_178103]^a^	hypothetical protein (34)	CJE1099 [YP_179092]^a^	hypothetical protein (43)
CJE0413 [YP_178432]	hypothetical protein (83)	CJE1795 [YP_179766]	membrane protein, putative (173)
CJE0761 [YP_178770]	putative periplasmic protein (182)	CJE1803 [YP_179773]	hypothetical protein (292)

^a ^– missing in some *C. jejuni *strains

**Table 6:** Proteins unique to *C. jejuni *and *C. coli*

Geneomic ID [Accession Number]	Geneomic ID [Accession Number]	Geneomic ID [Accession Number]	Geneomic ID [Accession Number]
CJE1150 [YP_179141]	CJE1098 [YP_179091]	CJE0425 [YP_178444]	CJE1131 [YP_179123]
CJE1153 [YP_179144]	CJE1101 [YP_179094]	CJE0387 [YP_178406]	CJE1375 [YP_179362]
CJE1154 [YP_179145]	CJE1093 [YP_179086]	CJE0388 [YP_178407]	CJE1376 [YP_179363]
CJE1125 [YP_179117]	CJE0835 [YP_178839]	CJE0389 [YP_178408]	CJE1392 [YP_179379]
CJE1126 [YP_179118]	CJE0690 [YP_178702]	CJE0266 [YP_178289]	CJE1550 [YP_179533]
CJE1104 [YP_179097]	CJE0671 [YP_178684]	CJE0053 [YP_178078]	CJE1551 [YP_179534]
CJE1105 [YP_179098]	CJE0477 [YP_178496]	CJE0067 [YP_178092]	CJE1552 [YP_179535]

**Table 7:** Proteins specific for *Campylobacter jejuni*

**Geneomic ID [Accession Number]**	**Geneomic ID [Accession Number]**	**Geneomic ID [Accession Number]**	**Geneomic ID [Accession Number]**
CJE0392 [YP_178411]*	CJE0213 [YP_178236]	CJE0257 [YP_178280]	CJE1053 [YP_179048]
CJE0602 [YP_178618]*	CJE0216 [YP_178239]	CJE0259 [YP_178282]	CJE1054 [YP_179049]
CJE0668 [YP_178681]*	CJE0223 [YP_178246]	CJE0267 [YP_178290]	CJE1424 [YP_179410]
CJE0669 [YP_178682]*	CJE0225 [YP_178248]	CJE0268 [YP_178291]	CJE1431 [YP_179417]
CJE1760 [YP_179732]*	CJE0239 [YP_178262]	CJE0271 [YP_178294]	CJE1432 [YP_179418]
CJE0204 [YP_178227]	CJE0240 [YP_178263]	CJE0574 [YP_178590]	CJE1433 [YP_179419]
CJE0205 [YP_178228]	CJE0245 [YP_178268]	CJE0946 [YP_178948]	CJE1470 [YP_179456]
CJE0206 [YP_178229]	CJE0247 [YP_178270]	CJE1046 [YP_179042]	CJE1629 [YP_179610]
CJE0208 [YP_178231]	CJE0248 [YP_178271]	CJE1048 [YP_179043]	CJE1829 [YP_179799]
CJE0211 [YP_178234]	CJE0253 [YP_178276]	CJE1052 [YP_179047]	CJE1840 [YP_179810]

The first 5 proteins marked by * are present in all *C. jejuni *strains (NCTC 11168, RM1221, HB93-13, 84-25, CF93-6, 260.94, 11168 and 81-176). The other proteins are missing in one or more strains.
