# Supplementary material for: Molecular signatures (unique proteins and conserved indels) that are specific for the epsilon proteobacteria (Campylobacterales)
Source: BMC Genomics. 2006 Jul 4;7:167. doi: 10.1186/1471-2164-7-167 (PMC1557499; doi:10.1186/1471-2164-7-167)
Supplement: Additional file 1 — Proteins that are Unique to Wolinella succinogenes DSM 1740. In BLASTp and PSI-BLAST searches, no significant similarity to these proteins was detected for any other protein. The identification numbers of these proteins in Wolinella succinogenes DMS 1740 genome, accession numbers, protein lengths and information regarding putative function, if known, are provided. [file 1471-2164-7-167-S1.doc]

**Additional file 1**

**Proteins that are unique to *Wolinella succinogenes***

| **Protein**  **ID no.** | Accession number | **Function (Length)** | **Protein ID no.** | Accession number | **Function (Length)** | **Protein ID no.** | Accession number | **Function (Length)** |
| --- | --- | --- | --- | --- | --- | --- | --- | --- |
| WS0008 | 34556467 | CYTB protein (163) | WS0663 | 34557078 | hypoth. (121) | WS1451 | 34557795 | hypoth. (86) |
| WS0010 | 34556469 | hypoth. (302) | WS0667 | 34557080 | hypoth. (74) | WS1457 | 34557801 | hypoth. (311) |
| WS0035 | 34556492 | hypoth. (61) | WS0672 | 34557085 | hypoth. (139) | WS1485 | 34557824 | hypoth. (89) |
| WS0048 | 34556503 | hypoth. (258) | WS0729 | 34557134 | hypoth. (122) | WS1573 | 34557898 | hypoth. (108) |
| WS0089 | 34556542 | hypoth. (177) | WS0732 | 34557137 | hypoth. (67) | WS1574 | 34557899 | hypoth. (105) |
| WS0100 | 34556552 | hypoth. (77) | WS0777 | 34557177 | hypoth. (166) | WS1593 | 34557917 | hypoth. (90) |
| WS0152 | 34556600 | hypoth. (72) | WS0782 | 34557181 | hypoth. (59) | WS1616 | 34557934 | hypoth. (96) |
| WS0182 | 34556629 | hypoth. (66) | WS0797 | 34557195 | hypoth. (170) | WS1619 | 34557936 | hypoth. (194) |
| WS0210 | 34556653 | hypoth. (51) | WS0833 | 34557229 | hypoth. (39) | WS1662 | 34557977 | hypoth. (115) |
| WS0215 | 34556658 | hypoth. (128) | WS0871 | 34557265 | hypoth. (101) | WS1735 | 34558045 | hypoth. (80) |
| WS0227 | 34556670 | hypoth. (81) | WS0887 | 34557277 | hypoth. (53) | WS1762 | 34558069 | hypoth. (70) |
| WS0247 | 34556687 | hypoth. (251) | WS0891 | 34557281 | hypoth. (71) | WS1820 | 34558120 | hypoth. (113) |
| WS0251 | 34556691 | hypoth. (246) | WS0893 | 34557283 | hypoth. (55) | WS1832 | 34558131 | hypoth. (161) |
| WS0261 | 34556701 | hypoth. (66) | WS0909 | 34557299 | hypoth. (137) | WS1854 | 34558152 | hypoth. (76) |
| WS0262 | 34556702 | hypoth. (65) | WS0957 | 34557345 | hypoth. (47) | WS1867 | 34558162 | hypoth. (214) |
| WS0268 | 34556707 | hypoth. (122) | WS0961 | 34557349 | hypoth. (131) | WS1888 | 34558183 | hypoth. (180) |
| WS0319 | 34556755 | hypoth. (99) | WS0980 | 34557368 | hypoth. (40) | WS1889 | 34558184 | hypoth. (229) |
| WS0364 | 34556797 | hypoth. (239) | WS1079 | 34557461 | hypoth. (86) | WS1929 | 34558223 | hypoth. (251) |
| WS0366 | 34556799 | hypoth. (149) | WS1083 | 34557464 | hypoth. (80) | WS1936 | 34558227 | hypoth. (246) |
| WS0334 | 34556769 | hypoth. (137) | WS1101 | 34557479 | hypoth. (132) | WS1963 | 34558251 | hypoth. (71) |
| WS0388 | 34556819 | hypoth. (62) | WS1151 | 34557523 | hypoth. (195) | WS1984 | 34558272 | hypoth. (60) |
| WS0406 | 34556835 | hypoth. (90) | WS1165 | 34557537 | hypoth. (61) | WS1993 | 34558280 | hypoth. (73) |
| WS0431 | 34556858 | hypoth. (204) | WS1204 | 34557572 | hypoth. (59) | WS2027 | 34558313 | hypoth. (101) |
| WS0500 | 34556923 | hypoth. (105) | WS1255 | 34557619 | hypoth. (68) | WS2028 | 34558314 | hypoth. (162) |
| WS0531 | 34556952 | hypoth. (135) | WS1282 | 34557646 | hypoth. (187) | WS2029 | 34558315 | hypoth. (186) |
| WS0532 | 34556953 | hypoth. (163) | WS1314 | 34557675 | hypoth. (68) | WS2032 | 34558318 | hypoth. (64) |
| WS0535 | 34556955 | hypoth. (122) | WS1363 | 34557716 | hypoth. (71) | WS2033 | 34558319 | hypoth. (241) |
| WS0539 | 34556959 | hypoth. (219) | WS1373 | 34557725 | hypoth. (64) | WS2034 | 34558320 | hypoth. (177) |
| WS0571 | 34556990 | hypoth. (173) | WS1393 | 34557744 | hypoth. (125) | WS2041 | 34558326 | hypoth. (180) |
| WS0625 | 34557041 | hypoth. (33) | WS1395 | 34557746 | hypoth. (76) | WS2053 | 34558338 | hypoth. (293) |
| WS0636 | 34557052 | hypoth. (105) | WS1437 | 34557783 | hypoth. (146) | WS2074 | 34558358 | hypoth. (125) |
| WS0639 | 34557055 | hypoth. (320) | WS1439 | 34557784 | hypoth. (141) | WS2101 | 34558384 | hypoth. (233) |
| WS0647 | 34557063 | hypoth. (151) | WS1446 | 34557791 | hypoth. (252) | WS2144 | 34558424 | hypoth. (146) |
| WS0659 | 34557074 | hypoth. (48) | WS1447 | 34557792 | hypoth. (106) | WS2218 | 34558491 | hypoth. (222) |

These proteins or ORFs do not show significant similarity to any other proteins in the databases.
